# Obesity and Health Risk Factors in Czech Artillery Soldiers

**DOI:** 10.1093/milmed/usaf600

**Published:** 2025-12-24

**Authors:** Jachym Simsa, Martin Musalek, Karel Sykora, Ivana Kinkorova, Jan Malecek, Michal Vagner, Libor Wawrzacz

**Affiliations:** Department of Military Physical Education, Faculty of Physical Education and Sport, Charles University, Jose Martiho 269/31, Prague 16252, Czech Republic; Department of Social Sciences Foundation in Kinanthropology, Faculty of Physical Education and Sport, Charles University, Jose Martiho 269/31, Prague 16252, Czech Republic; Department of Social Sciences Foundation in Kinanthropology, Faculty of Physical Education and Sport, Charles University, Jose Martiho 269/31, Prague 16252, Czech Republic; Department of Military Physical Education, Faculty of Physical Education and Sport, Charles University, Jose Martiho 269/31, Prague 16252, Czech Republic; Department of Adapted Physical Education and Sport Medicine, Faculty of Physical Education and Sport, Jose Martiho 269/31, Prague 16252, Czech Republic; Sport Sciences—Biomedical Department, Faculty of Physical Education and Sport, Charles University, Jose Martiho 269/31, Prague 16252, Czech Republic; Department of Military Physical Education, Faculty of Physical Education and Sport, Charles University, Jose Martiho 269/31, Prague 16252, Czech Republic; Department of Military Physical Education, Faculty of Physical Education and Sport, Charles University, Jose Martiho 269/31, Prague 16252, Czech Republic; Centre of Physical Education and Sport, University of Defence, Kounicova 65, Brno 66210, Czech Republic; Department of Military Internal Medicine and Military Hygiene, Military Faculty of Medicine, University of Defence, Trebesska 1575, Hradec Kralove 50001, Czech Republic

**Keywords:** BMI, body composition, body fat percentage, bone mineral density, Czech Soldiers, DXA, military, obesity, fat free mass, visceral adipose tissue

## Abstract

**Introduction:**

The physical fitness and body composition of soldiers are critical for operational readiness and overall health. Military service demands optimal levels of muscle strength, endurance, and cardiorespiratory fitness, alongside a healthy body fat percentage (%BF). However, unlike in the U.S. Armed Forces, Czech Soldiers are not required to meet specific %BF standards, and the prevalence of excessive fat remains unknown. Dual-energy X-ray absorptiometry (DXA) offers a precise and reliable assessment of body composition, yet its use in European military research remains limited. This study evaluates the body composition of Czech Artillery Soldiers using DXA, focusing on %BF, fat-free mass, visceral adipose tissue (VAT), and bone mineral density (BMD), compares %BF values with U.S. Army and civilian norms, and evaluates the practical implications of these findings for military health policy.

**Materials and Methods:**

A randomized cross-sectional study was conducted on 209 healthy adult active-duty Czech Artillery Soldiers (189 men, 20 women). Participants underwent a DXA scan to assess body composition using a Hologic Horizon system. Statistical analyses included the Mann-Whitney and Kruskal-Wallis tests, 1-way ANOVA, and generalized linear models (GLM) with age as a covariate. Health classifications were derived using BMI, %BF, and VAT thresholds for cardiometabolic risk and BMD T-scores for osteopenia and osteoporosis. The protocol adhered to the approval granted by the relevant institutional ethics committee (No. 246/2021).

**Results:**

Czech Artillery Soldiers and U.S. Soldiers had nearly identical %BF, 24.3% in Czech men vs. 24.3% in U.S. men; 33.8% vs. 33.3% in women, respectively. Overall, excessive %BF (≥25%) was observed in 41.2% of participants. Age group was a significant predictor of VAT (*ω*^2^ = 0.20, *df* = 6, *H* = 42.51, *P* < .001) and %BF (*ω*^2^ = 0.07, *df = *6, *H = *18.73, *P* < .005), with older participants exhibiting higher levels. High prevalence of osteopenia was found, reaching 20% in both sexes. Contrary to expectations, the GLM revealed that there are no significant differences among units of the Czech Artillery Brigade in any measured variable when age is considered.

**Conclusions:**

DXA-measured body composition in Czech Artillery Soldiers closely matches contemporary U.S. Army values. The high prevalence of overweight and osteopenia underscores the need for preventive and educational strategies addressing both metabolic and skeletal health risks. Future research should determine the prevalence of osteopenia and osteoporosis across the Czech Armed Forces to inform appropriate screening practices and targeted interventions.

## INTRODUCTION

Because of the demanding nature of military service, soldiers must sustain high standards of physical fitness, including cardiorespiratory fitness, muscle strength and endurance, and a high ratio of lean to fat mass. Physical fitness is essential not only for general health but also for the successful execution of military‑specific tasks.^1^ Fit soldiers experience fewer injuries and recover faster, directly supporting operational readiness.^2^ Poor fitness elevates the risk of cardiovascular disease^3^ and chronic conditions such as type II diabetes,^4^ hypertension,^5^ and obesity.^6^^,^^7^ A Czech civilian–military comparison from 2010 (*n = *5,511 civilians; *n = *9,622 soldiers) found that mandatory training in the military did not fully offset declines in strength, endurance, or cardiorespiratory fitness.^8^ Thus, the rising prevalence of obesity in both the general^9^ and military populations^10^ threatens the operational capabilities of the armed forces,^11^ and warrants closer attention.

Although Czech Soldiers are required to meet specific criteria based on physical fitness,^12^ there are no enforced standards for body fat percentage (%BF), unlike in the U.S. Armed Forces, where %BF requirements are regularly monitored.^13^ Within European militaries, body composition is commonly estimated using bioelectrical impedance^14^^,^^15^ or circumference‑based equations,^8^^,^^16^ methods that lack the precision of dual‑energy X‑ray absorptiometry (DXA).^17^ DXA provides reliable estimates of whole‑body and regional fat mass, lean mass, visceral adipose tissue (VAT), and bone mineral density (BMD), parameters directly relevant to injury risk, metabolic health, and long‑term readiness.^18^^,^^19^ However, European data on DXA‑derived body composition in soldiers remain scarce, limiting evidence‑based policy development and health monitoring.

The study describes the current body composition of the Czech Artillery Brigade personnel using DXA, with specific attention to %BF, VAT, and BMD. We compare our findings with U.S. Army reference values recently published by Taylor et al. (2024)^20^ and estimate the prevalence of overweight/obesity, metabolic syndrome risk (based on VAT thresholds), and low BMD (osteopenia/osteoporosis) using established cut-off values.^18^^,^^19^ In addition, we evaluate the agreement between DXA-derived %BF and conventional body mass index (BMI) categories.

Our goals were to (1) provide a precise body composition profile of Czech Artillery Soldiers, considering participants’ sex, age, and military unit affiliation; (2) contextualize Czech Artillery Soldiers’ data against large, contemporary military cohorts; and (3) evaluate the practical implications of body composition findings for military health policy, including the relevance of BMI as a screening tool. These insights can inform screening policies, targeted interventions, and future surveillance strategies to preserve operational effectiveness.

## METHODS

### Study Design

We conducted a cross-sectional study using stratified random sampling to ensure a representative sample across the Czech Artillery Brigade, minimizing selection bias and providing a comprehensive analysis of body composition. The protocol was approved by the Ethics Committee of the Faculty of Physical Education and Sport, Charles University, Czech Republic (No. 246/2021).

### Participants

Originally, 250 soldiers were assigned to a measurement session. Forty-one participants were not analyzed because of time constraints, temporary medical exemptions, or conflicting duties, yielding 209 analyzed participants. Women formed a small subset (*n = *20); therefore, gender‑specific inferential analyses were limited, and women were included only in descriptive summaries. Participant characteristics are presented in Table 1.

**Table 1.** Demographic and Body Composition Data

Variable	Men (*n = *189)					Women (*n = *20)				
	Mean	SD	Median	Min	Max	Mean	SD	Median	Min	Max
Age (years)	31.5	6.5	30.0	20.0	53.0	32.1	5.3	30.5	23.0	44.0
Height (cm)	179.6	6.8	179.1	160.7	196.1	166.4	5.5	168.2	156.2	173.6
Weight (kg)	85.3	12.7	84.1	51.5	132.4	68.1	12.4	64.7	54.2	99.6
BMI (kg/m^2^)	26.4	3.3	26.1	19.7	38.4	24.7	4.8	23.1	18.2	38.1
BF (%)	24.3	3.8	23.9	16.0	36.8	33.8	5.4	33.0	23.6	48.0
FFM (kg)	64.4	8.3	64.3	41.3	91.2	44.6	5.6	42.9	36.2	58.6
VAT (cm^2^)	77.0	24.0	70.8	43.9	192.0	51.0	30.4	39.7	28.1	145.0
BMD (T-score)	−0.2	0.9	−0.1	−2.7	2.5	0.3	1.0	0.7	−1.6	2.1

Abbreviations: BF = body fat, BMD = bone mineral density, BMI = body mass index, FFM = fat free mass, SD = standard deviation, VAT = visceral adipose tissue.

### Selection Process

All personnel of the Czech Artillery Brigade were screened for eligibility. Exclusion criteria included civilian staff, acute injury or illness, pregnancy, or other conditions contraindicating DXA screening. For Group analyses, participants were categorized into 3 cohorts based on their primary duties: (1) “military”—firing batteries, actively serving personnel engaged in field operations; (2) “support”—logistical and technical personnel; and (3) “staff”—administrative or command-level personnel. As the Czech Artillery Brigade represents the sole artillery formation within the Czech Armed Forces, this sampling covered all relevant operational categories within the branch. Operational deployments, training tasks, and medical appointments accounted for most non-participation. For security reasons, we do not disclose the exact number of the Brigade’s personnel or its units.

### Body Composition Analysis

A whole-body DXA scan was performed using a Hologic Horizon QDR-4500A X-ray Bone Densitometer (Hologic, Inc., Bedford, MA, USA). The NHANES BCA reference curves were applied to account for its biased measurement of lean mass.^21^ Each measurement day, the densitometer was calibrated using a manufacturer-provided calibration phantom. In accordance with the best practice guidelines for DXA measurement protocols in athletic populations,^22^ all measurements were conducted in the morning following an overnight fast. Participants were instructed to avoid alcohol consumption and intense physical activity for at least 24 hours before the assessment and to maintain a regular hydration routine over the previous 48 hours. On the morning of the assessment, they were asked to minimize fluid intake, and to void their bladder immediately beforehand. Scans were performed according to the manufacturer’s guidelines, with minimal clothing and all jewelry removed. All body scans were analyzed by a single, professionally trained staff member.

Although the limitations of BMI are well recognized,^23^ it remains a significant health-risk factor.^5^ Therefore, we categorized participants by BMI using WHO guidelines and compared these categories with those based on %BF, following Potter et al. (2024),^24^ who derived %BF thresholds using metabolic syndrome prevalence rates within BMI-defined categories. The %BF values were set at 25% and 30% for men, and 36% and 42% for women, representing the upper limits for overweight and obese classifications, respectively. These thresholds enabled us to align %BF classifications with metabolic health risks in a manner comparable to BMI-based categories.

To compare %BF with appropriate reference values, we analyzed age-, race-, and gender-matched percentiles from NHANES BCA reference curves integrated into the DXA APEX software. To classify participants’ cardiometabolic risk factors, we used cut-off points for VAT area, with thresholds set at 154 cm^2^ for men and 143 cm^2^ for women.^18^ To analyze bone health, we used BMD T-score cut-off points for osteopenia and osteoporosis. T-scores between −1 and −2.4 indicate osteopenia, and scores ≤−2.5 indicate osteoporosis.^19^

### Statistical Analysis

We assessed data normality for all dependent variables (BMI, %BF, FFM, VAT, BMD) using the D’Agostino-Pearson test. To investigate the degree of association between selected variables, we performed correlation analyses using Pearson’s *r* for parametric data and Spearman’s *ρ* for non-parametric data. To analyze differences among Groups (military, support, staff), we applied 1-way ANOVA if the variable met the assumptions, or the Kruskal-Wallis H test (K-W) if it did not, utilizing Holm’s correction in both methods. We used the same approach to analyze differences among age groups.

After initial analyses suggested significant differences in VAT among groups, we conducted a generalized linear model (GLM) analysis to verify whether these differences persisted after accounting for age. The GLM specified a gamma distribution and log link function to account for the skewed distribution of VAT. Additionally, we conducted a post hoc Monte Carlo simulation-based power analysis to examine the age effect in this model.

The statistical significance level was set at *α* = .05. For 1-way ANOVA, we used eta squared (*η*^2^) as the measure of effect size and Hays’ *ω*^2^ for the K-W test. The thresholds for small, medium, and large effect sizes were defined as 0.01, 0.06, and 0.12, respectively, for both *η*^2^ and Hays’ *ω*^2^.^25^

Descriptive statistics were performed using JASP ver. 0.18.3.^26^ All further analyses were performed using R ver. 4.4.2^27^ within the RStudio ver. 24.09.1,^28^ with the MASS package^29^ for GLM, the FSA package^30^ for Dunn’s post hoc test, and the fBasics package^31^ for D’Agostino-Pearson test.

## RESULTS

Data for VAT (*P* < .001), %BF (*P* < .05), and BMI (*P* < .001) did not follow a normal distribution. Group analyses revealed no significant differences among units in body composition, except for VAT (*P* < .05). After controlling for age, the GLM for VAT found no significant group effects, with “staff” showing a marginally higher VAT than the “military” (*P* = .061). The model revealed a positive association with age (*β* = .0189, 95% CI, 0.0129-0.0250, *P* < .001), corresponding to a 1.91% (95% CI 1.30%-2.53%) increase in VAT area per year, indicating that older soldiers in our sample may be at increased risk of developing metabolic syndrome. Power analysis of this model exceeded 99% at *α = *.05. These results suggest that occupational role does not independently influence any of the measured variables within the Czech Artillery Brigade.

Age group analyses of %BF (*ω*^2^ = 0.07, *df = *6, *H = *18.73, *P* < .005) and VAT (*ω*^2^ = 0.20, *df = *6, *H = *42.51, *P* < .001) revealed moderate to large effects. After applying Holm’s correction, differences in BMI were insignificant. Post hoc differences of %BF and VAT are detailed in Tables 2 and 3, respectively.

**Table 2.** Men %BF by Age Group

Age group (years)	*n*	**BF (%)**				
		Mean	Median	SD	Min	Max
20-24	24	23.3	23.2	3.8	16.9	33.0
25-29^a^	55	23.3	22.9	3.9	17.1	36.0
30-34	63	24.9	24.4	3.6	16.0	34.2
35-39	25	24.8	25.7	3.1	16.5	29.8
40-44^a^	13	27.5	26.8	4.5	19.7	36.8
45-49	5	23.2	22.8	2.2	21.0	26.7
50-54	4	23.0	23.1	0.8	22.0	23.7

*P* < .05, *P*-values were adjusted using Holm’s correction.

Abbreviations: BF = body fat, SD = standard deviation.

a Dunn’s post-hoc comparison.

**Table 3.** Men VAT area by Age Group

Age group (years)	*n*	VAT (cm^2^)				
		Mean	Median	SD	Min	Max
20-24^a^ ^,^ ^b^ ^,^ ^c^ ^,^ ^d^	24	63.0	60.8	11.3	50.2	94.5
25-29^e^	55	71.6	67.6	22.1	43.9	192.0
30-34^a^ ^,^ ^f^	63	74.6	72.5	17.4	44.6	130.0
35-39^b^	25	83.7	81.0	23.5	51.6	133.0
40-44^c^ ^,^ ^e^ ^,^ ^f^	13	118.7	120.0	34.1	61.7	170.0
45-49	5	75.8	72.8	9.7	67.1	92.3
50-54^d^	4	95.6	93.6	17.6	77.1	118.0

a,d *P* < .05 = Dunn’s post-hoc comparison, *P*-values were adjusted using Holm’s correction.

b,f *P* < .01 = Dunn’s post-hoc comparison, *P*-values were adjusted using Holm’s correction.

c,e *P* < .001 = Dunn’s post-hoc comparison, *P*-values were adjusted using Holm’s correction.

Overweight and obesity were prevalent. Based on BMI, 50.2% of men and 25% of women in our sample were overweight (BMI 25.0-29.9), and 10.1% of men and 15% of women were obese (BMI ≥ 30). Conversely, BMI failed to identify excessive %BF in 17.4% of participants. Using %BF thresholds instead of BMI, nearly half of those classified as overweight or obese were more accurately categorized into lower adiposity ranges. Overall, the observed prevalence of overweight and obesity based on %BF was 34.5% and 6.7%, respectively, in both sexes combined, using the gender-specific thresholds defined earlier. Based on NHANES BCA reference curves, 95.7% of participants fell between the 5th and 85th age-, race-, and gender-matched %BF percentiles. The median percentile was 37, ranging from 3 to 92, with an interquartile range of 28.

Additionally, correlation analyses showed considerably lower correlation between %BF and BMI (*r *= 0.62, *ρ*  =  0.56; *P* < .001) compared that observed in NHANES data for white men of the same age (*r *= 0.80-0.85; *P* < .05),^32^ and only a weak correlation between %BF and age in our sample (*ρ*  =  0.22, *P* < .05). A moderate correlation was observed between BMD and %BF (*ρ* = −0.30, *P* < .001), as well as between age and VAT (*ρ*  =  0.44, *P* < .001), and between BMD and FFM (*r *= 0.297, *P* < .001). No significant correlation was found between age and either BMD or BMI.

Based on VAT area measurements, we identified 1 woman (5%) and 5 men (2.1%) who were at increased risk of developing metabolic syndrome.^18^ Regarding bone health, 20% of women and 19.6% of men had osteopenic BMD T-scores, while only 1 participant (a man) met the osteoporosis threshold. Notably, none of the women had gone through menopause, suggesting that their reduced bone density may have been influenced by other factors, such as eating habits, possible variations in estrogen levels, or menstrual irregularities.^33^

## DISCUSSION

This study fills a critical gap in European military medicine by providing DXA‑based body composition data for Czech Artillery Soldiers. Our findings mirror recent U.S. Army data, with nearly identical %BF values between cohorts despite geographic and cultural differences. To provide a single summary value for comparison, pooled values were calculated across both cohorts of the original study by Taylor et al. (2024),^20^ resulting in a mean %BF of 24.3 ± 6.5 in men (*n = *2012) and 33.3 ± 6.3 in women (*n = *963). These values closely align with our sample of Czech Artillery Soldiers (24.3 ± 3.8%BF in men and 33.8 ± 5.4%BF in women). This convergence supports the applicability of U.S. Army reference values as a contextual benchmark for Central European forces. That being said, our study was conducted within a single military setting, and further data from other units would strengthen the regional applicability of these findings.

Our analyses revealed no significant association between specific military units and body composition within the Czech Artillery Brigade. Instead, age emerged as the primary predictor of VAT among Czech Artillery Soldiers, with our GLM analysis indicating a 1.91% (95% CI, 1.30%, 2.53%) increase in VAT area per year of age. The association between age and VAT was robust, as confirmed by our post hoc power analysis (99.8% power to detect the observed effect at *α = *0.05), and aligns with previous research linking increasing age to higher visceral fat and greater cardiometabolic risk.^34^ Although older soldiers showed higher VAT, we identified only 4 participants at elevated cardiometabolic risk. Interestingly, VAT appeared to decline steeply in age groups 45-49 (*n = *5) and 50-54 (*n = *4). We suggest that this observation is likely attributable to random variation, given the sample size in older age groups. This interpretation is supported by other research, which indicates that VAT area tends to increase consistently with age.^34^ However, the detection of individuals above VAT risk thresholds argues for cardiometabolic screening and targeted interventions.

The prevalence of osteopenia in both sexes, despite a relatively young age of the cohort, highlights the need to emphasize bone health within military health promotion programs, including regular resistance training and adequate calcium and vitamin D intake. Vitamin D deficiency, defined as a serum 25-hydroxyvitamin D level of <50 nmol/L, is widespread across all age groups in the Czech population and may impair bone metabolism and contribute to broader metabolic dysfunction, including obesity.^35^ Vitamin D and calcium supplementation has also been linked to a reduction in stress fractures in military settings,^36^ underscoring its importance for training continuity and maintaining operational readiness.

Although the limitations of BMI are well recognized, it remains useful for population-level comparisons and for benchmarking against civilian and international military data. In our sample, BMI indicated approximately 10% lower obesity prevalence than the Czech general population,^37^ yet its correlation with %BF (*r* = 0.62) was notably weaker than that observed in NHANES men of similar age (*r* = 0.80-0.85; *P* < .05), underscoring BMI’s limited accuracy in muscular military populations where lean mass can mask true adiposity. Although DXA provides precise body-composition estimates, it is impractical for large-scale or routine screening. In operational contexts, circumference-based indices, such as abdomen, hip, or waist circumference, offer practical and equipment-free alternatives that reflect adiposity and cardiometabolic risk.^20^ Incorporating such field-friendly metrics could improve health surveillance and inform evidence-based fitness policies.

Our findings underscore the complexity of body composition in military personnel and highlight the importance of accurate health assessments within military populations. Czech Artillery Soldiers exhibit body composition profiles remarkably similar to those of the U.S. Army, with near-identical %BF. The consistent body composition across units within the Brigade suggests that occupational role alone does not drive differences in obesity or body composition. Moreover, the significant age-related effects on %BF and VAT emphasize the need to consider age in body composition evaluations, as older soldiers showed increased risk factors for conditions like metabolic syndrome.

### Practical Implications

The findings of our study directly informed participating soldiers, each of whom received an individual consultation with a healthcare professional to discuss potential changes in eating habits, dietary supplementation, training, and lifestyle. Beyond that, our results suggest several actionable strategies for military health and readiness programs.

The observed prevalence of osteopenia in our sample represents an early warning signal and warrants systematic investigation in larger, representative cohorts across the Czech Armed Forces. Such evidence is essential for developing and implementing practical preventive strategies, including nutritional interventions (e.g., optimization of calcium and vitamin D intake). Evidence from randomized controlled trials in U.S. Military recruits demonstrates that daily supplementation with calcium (2,000 mg) and vitamin D (800-1,000 IU) can improve BMD,^38^ and reduce the incidence of stress fractures.^36^ Given the high prevalence of vitamin D deficiency in the Czech population,^35^ periodic screening of serum 25-hydroxyvitamin D levels should also be considered as part of routine military health monitoring to identify at-risk individuals. In addition to supplementation, structured education on bone health covering dietary sources of calcium, vitamin D, and safe sunlight exposure should be incorporated into existing health-promotion programs. Beyond nutritional measures, incorporating progressive strength and high-impact training can help reduce overuse injuries, including stress fractures, in physically active soldiers.^39^

Given the observed association between age and increased VAT, preventive efforts should focus on reducing long-term cardiometabolic risks through improved lifestyle education. Military health programs could incorporate more comprehensive information on dietary strategies for reducing visceral fat and preventing obesity, emphasizing balanced energy intake, higher protein quality, reduced consumption of ultra-processed foods, and improved meal timing during deployments or shift work. Nutritional education should be supported by practical measures, such as adjusting menu options in military dining facilities to include lower-energy yet nutrient-dense meals and clear labeling of healthier choices. Periodic workshops or consultations with nutrition specialists could further enhance soldiers’ understanding of how diet composition influences adiposity accumulation.

Collectively, these measures, from bone health promotion and targeted supplementation to improved nutritional guidance aimed at reducing VAT and obesity, could enhance soldiers’ metabolic and skeletal health alike, thereby reinforcing long-term operational readiness and resilience.

### Study Limitations

We sampled a substantial portion of the Czech Artillery Brigade; however, our findings may not fully represent the entire Czech Armed Forces or the Czech Army. The small number of women restricted our ability to conduct gender-specific analyses, and the relatively small sample size in older age groups limits the generalizability of results for older soldiers even within the Czech Artillery Brigade. The U.S. reference study by Taylor et al. (2024)^20^ did not report detailed DXA testing conditions (e.g., fasting status, hydration, or bladder voiding) and employed a different DXA system (GE Lunar iDXA, GE Healthcare), which limits direct comparability and should be considered when interpreting our results. Finally, the possibility of selection bias cannot be ruled out. Although non-participation was primarily because of other military duties during data collection, it is possible that those who did not take part differ in meaningful ways from those who did, potentially influencing the findings.

## CONCLUSIONS

This study provides a detailed evaluation of body composition in Czech Artillery Soldiers using DXA, revealing that nearly one-fifth of participants, irrespective of sex, were classified as osteopenic, raising concerns about bone health and injury susceptibility and highlighting the need for preventive strategies, particularly given the physical demands of military service. Along with the observed 41.2% incidence of overweight and obesity based on %BF, our results underscore the importance of combining precise body composition assessments with preventive and educational strategies within routine military health programs to address both metabolic and skeletal health risks. Contrary to expectations, no significant differences in body composition were found among the military, support, and staff groups.

Future research should focus on validating practical methods suitable for large-scale screening, such as circumference-based approaches, in the Czech Armed Forces, and should also examine longitudinal trends in body composition and evaluate the effectiveness of preventive measures across different military settings to support evidence-based health strategies.

## Data Availability

The data underlying this article will be shared on reasonable request to the corresponding author.

## INSTITUTIONAL CLEARANCE

Institutional clearance approved. Included in the approval of the Ethics Committee of the Faculty of Physical Education and Sport, Charles University, Czech Republic, no. 246/2021.

## References

[1] Center for Army Lessons Learned. Holistic Health and Fitness Handbook. U.S. Army Combined Arms Center; 2023. Accessed August 1, 2025. https://api.army.mil/e2/c/downloads/2023/06/05/25e44ff1/23-06-784-holistic-health-and-fitness-handbook-jun-23-public-release-1.pdf

[2] Knapik JJ. The importance of physical fitness for injury prevention: part 1. J Spec Oper Med. 2015;15:123–7. 25770810

[3] Timpka S , Petersson IF, Zhou C, Englund M. Muscle strength in adolescent men and risk of cardiovascular disease events and mortality in Middle age: a prospective cohort study. BMC Med. 2014;12:62. 10.1186/1741-7015-12-62 24731728 PMC4006633

[4] Crump C , Sundquist J, Winkleby MA, Sieh W, Sundquist K. Physical fitness among Swedish military conscripts and Long-Term risk for type 2 diabetes mellitus: a cohort study. Ann Intern Med. 2016;164:577–84. 10.7326/M15-2002 26954518 PMC4861045

[5] Crump C , Sundquist J, Winkleby MA, Sundquist K. Interactive effects of physical fitness and body mass index on the risk of hypertension. JAMA Intern Med. 2016;176:210–6. 10.1001/jamainternmed.2015.7444 26784837 PMC4803286

[6] Telford RD. Low physical activity and obesity: causes of chronic disease or simply predictors? Med Sci Sports Exerc. 2007;39:1233–40. 10.1249/mss.0b013e31806215b7 17762355

[7] Henriksson P , Shiroma EJ, Henriksson H, et al. Fit for life? Low cardiorespiratory fitness in adolescence is associated with a higher burden of future disability. Br J Sports Med. 2021;55:128–9. 10.1136/bjsports-2020-102605 32816793 PMC10207998

[8] Soumar L , Oberman C. Long-term monitoring of actual health status parameters of Czech population with emphasis on ACR servicemen. Vojenské Rozhledy. 2010;19-51:174–89.

[9] Williams EP , Mesidor M, Winters K, Dubbert PM, Wyatt SB. Overweight and obesity: prevalence, consequences, and causes of a growing public health problem. Curr Obes Rep. 2015;4:363–70. 10.1007/s13679-015-0169-4 26627494

[10] Knapik JJ , Redmond JE, Grier TL, Sharp MA. Secular trends in the physical fitness of United States army infantry units and infantry soldiers, 1976–2015. Mil Med. 2018;183:e414–e426. 10.1093/milmed/usx093 29447398

[11] American Security Project. Combating Military Obesity: Stigma’s Persistent Impact on Operational Readiness. American Security Project; 2023. Accessed July 9, 2024. http://www.jstor.org/stable/resrep53514

[12] Ministerstvo obrany ČR. Normativní výnos Ministerstva obrany č. 12/2011: Služební tělesná výchova v rezortu Ministerstva obrany. Published online 2011.

[13] Department of the Army. The Army Body Composition Program (AR 600-9). Published online July 2019. https://www.armyresilience.army.mil/abcp/pdf/ARN7779_AR600-9_FINAL.pdf

[14] Carretero-Krug A , Úbeda N, Velasco C, et al. Hydration status, body composition, and anxiety status in aeronautical military personnel from Spain: a cross-sectional study. Military Med Res. 2021;8:35. 10.1186/s40779-021-00327-2 PMC8170814 34074350

[15] Mikkola I , Keinänen-Kiukaanniemi S, Jokelainen J, et al. Aerobic performance and body composition changes during military service. Scand J Prim Health Care. 2012;30:95–100. 10.3109/02813432.2012.649631 22643154 PMC3378011

[16] Vantarakis A , Vezos N, Karakatsanis K, et al. The effects of exercise during a 10-week basic military training program on the physical fitness and the body composition of the Greek naval cadets. Mil Med. 2022;187:e1396–e1402. 10.1093/milmed/usab146 33876215

[17] Combest TM , Khan J, Tufano JJ, et al. Comparison of four body composition methods: circumference measurements, eight-point bioelectrical impedance analysis up to 500 and 1000 kHz to dual-energy X-ray absorptiometry to measure body fat percentage. Mil Med. 2025;190:e642–e648. 10.1093/milmed/usae439 39292528

[18] Katzmarzyk PT , Greenway FL, Heymsfield SB, Bouchard C. Clinical utility and reproducibility of visceral adipose tissue measurements derived from dual-energy X-ray absorptiometry in white and African American adults: visceral fat thresholds. Obesity (Silver Spring). 2013;21:2221–4. 10.1002/oby.20519 23794256 PMC3819404

[19] World Health Organization. Guidelines for Preclinical Evaluation and Clinical Trials in Osteoporosis. World Health Organization; 1998.

[20] Taylor KM , Castellani MP, Bartlett PM, Oliver TE, McClung HL. Development and cross-validation of a circumference-based predictive equation to estimate body fat in an active population. Obes Sci Pract. 2024;10:e747. 10.1002/osp4.747 38646612 PMC11026907

[21] Schoeller DA , Tylavsky FA, Baer DJ, et al. QDR 4500A dual-energy X-ray absorptiometer underestimates fat mass in comparison with criterion methods in adults. Am J Clin Nutr. 2005;81:1018–25. 10.1093/ajcn/81.5.1018 15883424

[22] Nana A , Slater GJ, Stewart AD, Burke LM. Methodology review: using dual-energy X-ray absorptiometry (DXA) for the assessment of body composition in athletes and active people. Int J Sport Nutr Exerc Metab. 2015;25:198–215. 10.1123/ijsnem.2013-0228 25029265

[23] Prentice AM , Jebb SA. Beyond body mass index. Obes Rev. 2001;2:141–7. 10.1046/j.1467-789x.2001.00031.x 12120099

[24] Potter AW , Chin GC, Looney DP, Friedl KE. Defining overweight and obesity by percent body fat instead of body mass index. *J Clin Endocrinol Metab*. 2024;110:e1103–7. 10.1210/clinem/dgae341 38747476 PMC11913102 38747476

[25] Lakens D. Calculating and reporting effect sizes to facilitate cumulative science: a practical primer for t-tests and ANOVAs. Front Psychol. 2013;4:863. 10.3389/fpsyg.2013.00863 24324449 PMC3840331

[26] JASP Team. JASP (Version 0.18.3). JASP Team; 2024. https://jasp-stats.org/

[27] R Core Team. R: A Language and Environment for Statistical Computing. R Foundation for Statistical Computing; 2024. https://www.R-project.org/

[28] RStudio Team. RStudio: Integrated Development Environment for R. RStudio, PBC; 2024. http://www.rstudio.com/

[29] Venables WN , Ripley BD. Modern Applied Statistics with S. 4th ed. Springer; 2002. https://www.stats.ox.ac.uk/pub/MASS4/

[30] Ogle DH , Doll JC, Wheeler AP, Dinno A. FSA: Simple Fisheries Stock Assessment Methods [R package]. R Foundation for Statistical Computing, Vienna, Austria; 2023. https://CRAN.R-project.org/package=FSA

[31] Wuertz D , Setz T, Chalabi Y, Boshnakov GN. fBasics: Rmetrics—Markets and Basic Statistics [R package]. R Foundation for Statistical Computing, Vienna, Austria; 2024. https://CRAN.R-project.org/package=fBasics

[32] Jeong SM , Lee DH, Rezende LFM, Giovannucci EL. Different correlation of body mass index with body fatness and obesity-related biomarker according to age, sex and race-ethnicity. Sci Rep. 2023;13:3472. 10.1038/s41598-023-30527-w 36859451 PMC9977890

[33] Nattiv A , Loucks AB, Manore MM, et al. The female athlete triad. Med Sci Sports Exerc. 2007;39:1867–82. 10.1249/mss.0b013e318149f111 17909417

[34] Machann J , Thamer C, Schnoedt B, et al. Age and gender related effects on adipose tissue compartments of subjects with increased risk for type 2 diabetes: a whole body MRI/MRS study. MAGMA. 2005;18:128–37. 10.1007/s10334-005-0104-x 16001284

[35] Holmannova D , Borsky P, Kremlacek J, et al. High prevalence of low vitamin D status in the Czech Republic: a retrospective study of 119,925 participants. Eur J Clin Nutr. 2025;79:641–52. 10.1038/s41430-025-01587-0 40033138 PMC12274130

[36] Lappe J , Cullen D, Haynatzki G, Recker R, Ahlf R, Thompson K. Calcium and vitamin D supplementation decreases incidence of stress fractures in female navy recruits. J Bone Miner Res. 2008;23:741–9. 10.1359/jbmr.080102 18433305

[37] Overweight and obesity—BMI statistics. Accessed July 12, 2024. https://ec.europa.eu/eurostat/statistics-explained/index.php? title=Overweight_and_obesity_-_BMI_statistics

[38] Gaffney-Stomberg E , Lutz LJ, Rood JC, et al. Calcium and vitamin D supplementation maintains parathyroid hormone and improves bone density during initial military training: a randomized, double-blind, placebo controlled trial. Bone. 2014;68:46–56. 10.1016/j.bone.2014.08.002 25118085

[39] Tingelstad HC , Robitaille E, O’Leary TJ, Laroche M-A, Larsen P, Reilly T. MSKI reduction strategies: evidence-based interventions to reduce musculoskeletal injuries in military service members. BMJ Mil Health. 2025;171:418–22. 10.1136/military-2024-002747 PMC12505056 39209759

